# Network-based analysis of comorbidities risk during an infection: SARS and HIV case studies

**DOI:** 10.1186/1471-2105-15-333

**Published:** 2014-10-24

**Authors:** Mohammad Ali Moni, Pietro Liò

**Affiliations:** Computer Laboratory, University of Cambridge, William Gates Building, 15 JJ Thomson Avenue, Cambridge, CB3 0FD UK; Department of Computer Science & Engineering, Pabna University of Science & Technology, Pabna, Bangladesh

**Keywords:** Comorbidities infections, Disease associations, SARS, HIV

## Abstract

**Background:**

Infections are often associated to comorbidity that increases the risk of medical conditions which can lead to further morbidity and mortality. SARS is a threat which is similar to MERS virus, but the comorbidity is the key aspect to underline their different impacts. One UK doctor says "I’d rather have HIV than diabetes" as life expectancy among diabetes patients is lower than that of HIV. However, HIV has a comorbidity impact on the diabetes.

**Results:**

We present a quantitative framework to compare and explore comorbidity between diseases. By using neighbourhood based benchmark and topological methods, we have built comorbidity relationships network based on the OMIM and our identified significant genes. Then based on the gene expression, PPI and signalling pathways data, we investigate the comorbidity association of these 2 infective pathologies with other 7 diseases (heart failure, kidney disorder, breast cancer, neurodegenerative disorders, bone diseases, Type 1 and Type 2 diabetes). Phenotypic association is measured by calculating both the Relative Risk as the quantified measures of comorbidity tendency of two disease pairs and the *ϕ*-correlation to measure the robustness of the comorbidity associations. The differential gene expression profiling strongly suggests that the response of SARS affected patients seems to be mainly an innate inflammatory response and statistically dysregulates a large number of genes, pathways and PPIs subnetworks in different pathologies such as chronic heart failure (21 genes), breast cancer (16 genes) and bone diseases (11 genes). HIV-1 induces comorbidities relationship with many other diseases, particularly strong correlation with the neurological, cancer, metabolic and immunological diseases. Similar comorbidities risk is observed from the clinical information. Moreover, SARS and HIV infections dysregulate 4 genes (ANXA3, GNS, HIST1H1C, RASA3) and 3 genes (HBA1, TFRC, GHITM) respectively that affect the ageing process. It is notable that HIV and SARS similarly dysregulated 11 genes and 3 pathways. Only 4 significantly dysregulated genes are common between SARS-CoV and MERS-CoV, including NFKBIA that is a key regulator of immune responsiveness implicated in susceptibility to infectious and inflammatory diseases.

**Conclusions:**

Our method presents a ripe opportunity to use data-driven approaches for advancing our current knowledge on disease mechanism and predicting disease comorbidities in a quantitative way.

**Electronic supplementary material:**

The online version of this article (doi:10.1186/1471-2105-15-333) contains supplementary material, which is available to authorized users.

## Background

The term "comorbidity" refers to the coexistence of multiple diseases or disorders in relation to a primary disease or disorder in an individual [1]. A comorbidity relationship between two diseases exists whenever they appear simultaneously in a patient more than chance alone [2]. It represents the co–occurrence of diseases or presence of different medical conditions one after another in the same patient [1, 3]. Some diseases or infections can coexist in an individual by coincidence, and there is no pathological association among them. However, in most of the cases, multiple diseases (acute or chronic events) occur together in a patient because of the associations among them. These comorbidity associations can be due to direct or indirect causal relationships and the shared risk factors among diseases [4]. For an instance, a type of genetic abnormality linked to cancer is more common in patient of type 2 diabetes than other people [5]. Examples of comorbidity studies are many, often referring to chronic obstructive pulmonary disease (COPD) [6, 7], obesity [8], mental disorders [9], immune-related diseases [10], cancer [11] etc.

Comorbidity can be attributed to the disease connections on the molecular level, such as dysregulated genes, PPIs (protein–protein interactions), and metabolic pathways as potential causes of comorbidity [1, 3, 12, 13]. From a genetic perspective, a pair of diseases is connected because they have both been associated with the same dysregulated genes [14, 15], whereas from a proteomics perspective phenotypically similar diseases are related via biological modules such as PPIs or molecular pathways [16, 17].

Population-based disease association is important in conjunction with molecular and genetic data to uncover the molecular origins of diseases and disease comorbidities. Patient medical records contain important clarification regarding the co-occurrences of diseases affecting the same patient [2]. During the last few years, several researchers have been conducted the disease comorbidity analysis to understand the origins of many diseases [1, 12, 18]. Goh, Cusick, Valle, Childs, Vidal, Barabasi et al. and Feldman, Rzhetsky, Vitkup et al. built networks of gene-disease associations by connecting diseases that have been associated with the same genes [14, 15], whereas Lee, Park, Kay, Christakis, Oltvai and Barabási et al. constructed a network in which two diseases are linked if metabolic reactions are associated between them [13]. Disease association studies from proteomic point of view have been studied by Rual, Venkatesan, Hao, Hirozane-Kishikawa, Dricot, Li, Berriz, Gibbons, Dreze, Ayivi-Guedehoussou et al. and Stelzl, Worm, Lalowski, Haenig, Brembeck, Goehler, Stroedicke, Zenkner, Schoenherr, Koeppen et al. [19, 20]. Rzhetsky, Wajngurt, Park and Zheng et al. inferred the comorbidity links between 161 disorders from the disease history of 1.5 million patients [12]. However, all of these efforts have focused on the role of a single molecular or phenotypic measure to capture disease–disease relationships. In our work we have used disease–gene associations, PPIs, molecular pathways and clinical information to obtain statistically significant associations and comorbidity risks among diseases.

Inflammation is a hallmark of many serious human infectious diseases associated to a wide variety of infections, such as HIV-1 [21]. UK doctor Max Pemberton says "I’d rather have HIV than diabetes" as life expectancy among diabetes patients is lower than that of HIV [22]. However, HIV has a comorbidity impact on the diabetes. Also the flu can cause complications, including bacterial pneumonia, or the worsening of chronic health problems. Asthma is the most common comorbidity in patients hospitalized for swine influenza (H1N1) infection [23]. Dengue can cause myocardial impairment, arrhythmias and, occasionally, fulminant myocarditis [24]. Chronic medical conditions, such as heart disease, lung disease, diabetes, renal disease, rheumatologic disease, dementia, and stroke are risk factors for influenza complications [25]. Common chronic infections such as periodontitis or infection with Helicobacter pylori may also increase stroke risk [26]. Moreover, the severity of pneumonia in patients coinfected with influenza virus and bacteria is significantly higher than in those infected with bacteria alone. The incidence of flu is higher in children and younger adults than in older individuals, but influenza-associated morbidity and mortality increase with age, especially for individuals with underlying medical conditions such as chronic cardiovascular diseases [27]. During the ageing process the immune system becomes compromised and it causes an increasing inflammation [28]. In particular, chronic inflammation (inflammageing) and metabolic function are strongly affected by the ageing process [29]. The ageing of populations is leading to an unprecedented increase different diseases like cancer and fatalities. It is reported that 80% of the elderly population has three or more chronic conditions [30].

On the other hand, respiratory viruses are an emerging threat to global health security and have led to worldwide epidemics with substantial morbidity and mortality [31]. Coronaviruses (CoVs) cause respiratory and enteric diseases in human and other animals that induce fatal respiratory, gastrointestinal and neurological disease. Severe acute respiratory syndrome (SARS) is an epidemic human disease, is caused by a coronavirus (CoV), called SARS-associated coronavirus (SARS-CoV) [32]. SARS patients may present with a spectrum of disease severity ranging from flu-like symptoms and viral pneumonia to acute respiratory distress syndrome and death [33]. Most of the deaths were attributed to complications related to sepsis, ARDS and multiorgan failure, which occurred commonly in the elderly for comorbidities [34]. Age and comorbidity (e.g. diabetes mellitus, heart disease) were consistently found to be significant independent predictors of various adverse outcomes in SARS [35]. Children with SARS have better prognosis than adults [34]. Advanced age and comorbidities were significantly associated with increased risk of SARS-CoV related death, due to acute respiratory distress syndrome [35]. Mild degree of anaemia is common in the SARS infected patients and patients who have recovered from SARS show symptoms of psychological trauma [34]. Another novel coronavirus MERS-CoV, which is a new threat for public health, has similar clinical characteristics to SARS-CoV, but the comorbidity is the key aspect to underline their different impacts [36, 37]. MERS-CoV causes respiratory infections of varying severity and sometimes fatal infections in humans including kidney failure and severe acute pneumonia [38]. Despite sharing some clinical similarities with SARS (eg, fever, cough, incubation period), there are also some important differences such as the rapid progression to respiratory failure, which we have studied on comorbidities point of view.

Infection with the human immunodeficiency virus-1 (HIV) and the resulting acquired immune deficiency syndrome (AIDS) affects cellular immune regulation [39]. HIV infection severely impacts on the immune system causing phenotypic changes in peripheral cells and dysregulates the innate immune system [40]. Significant number of HIV-1 infected patients exhibits osteopenia and osteoporosis, leading to higher incidence to develop weak and fragile bones during the course of disease [41]. HIV has also been associated with an increased risk of developing both diabetes and cardiovascular disease [42]. Infection with HIV weakens the immune system and reduces the body’s ability to fight infections that may lead to cancer [43, 44]. People infected with human immunodeficiency virus (HIV) have a higher risk of some types of cancer (Kaposi sarcoma, non-Hodgkin lymphoma, cervical cancer, anal, liver, lung cancer, and Hodgkin lymphoma) than uninfected people [45]. Many people infected with HIV are also infected with other viruses that cause certain cancers [46, 47]. HIV infection even when controlled by highly active antiretroviral therapy (HAART) is being linked to chronic inflammation [48]. People with HIV-1 infection appear to have a markedly higher rate of chronic kidney disease than the general public [49]. It is because some of the risk factors associated with HIV-1 acquisition are the same as those that lead to kidney disease because of the virus itself and some therapies (e.g. HAART therapy). Antiretroviral therapy for HIV may increase the risk of developing metabolic syndrome (abdominal obesity, hyperglycaemia, dyslipidaemia and hypertension) and thus predispose to type 2 diabetes and cardiovascular disease. Many of the biologic factors thought to be causally associated with inflammation in HIV disease are also thought to be causally associated with the inflammation of ageing [50].

Infections (acute and chronic conditions) are often associated to comorbidity that increases the risk of medical conditions which can lead to further morbidity and mortality. Comorbidities related to flu have been recently investigated [51]. Comorbidities for tuberculosis have also been studied recently [52, 53]. To understand the overall mechanism we have studied the comorbidity associations of SARS and HIV infections. Both HIV and SARS are emerging infectious diseases in the modern world; each of these diseases has caused global societal and economic impact related to unexpected illnesses and deaths [54]. SRAS is a significant public health threat and HIV is a long term chronic infection. Since these two infections are associated with high mortality rates and there are no clinically approved antiviral treatments or vaccines available for either of these infections, we have selected these two infections for our study. Centred on the SARS and HIV-1 infections we have investigated highly heterogeneous disease comorbidity networks using the disease–gene associations, PPI subnetwork, molecular pathways and clinical information.

## Results and discussion

### Results

We have presented a systematic and quantitative approach to discover human disease comorbidities using different sources of available mRNA expression, protein-protein interactions, signalling pathways, disease–gene associations, disease–disease associations and disease–drug associations data. It has been shown that SARS coronavirus infects and replicates in a wide variety of host cells in susceptible animals and human beings [55, 56]. To understand the host response to this pathogen, we analysed the gene expression patterns of SARS infected patients, compared to normal subjects using oligo-nucleotide microarrays from the NCBI GEO ( http://www.ncbi.nlm.nih.gov/geo/query/acc.cgi?acc=GSE1739) [55]. We analysed the microarray gene expression data of over 8,700 genes from the PBMCs of 10 SARS patients, and compared with healthy control samples. We found that 274 genes (*p* < 0.01, > 1.5 fold change) were differentially expressed as compared to healthy controls in which 120 genes were significantly up regulated and 154 genes were significantly down regulated (see Additional file 1: Table S1).

On the other hand, monocytes are the key immune responsive cells whose function is adversely impacted by HIV-1. HIV-1 infection radically alters the monocyte phenotype, which is reflected in an HIV-1 induced gene expression analysis. Monocyte gene expression microarray data were collected for control and HIV patients from the NCBI GEO ( http://www.ncbi.nlm.nih.gov/geo/query/acc.cgi?acc=GSE18464) [57]. To find out the significant dysregulated genes during the HIV-1 infection, we have performed global gene expression analysis. We found that 186 genes (*p* < 0.01, > 1.5 fold change) were differentially expressed as compared to healthy controls in which 71 genes were up regulated and 115 genes were down regulated (see Additional file 2: Table S2).

Considering the significantly dysregulated genes of SARS (274 genes) and HIV-1 (186 genes) infections, and gene-disease associations information, we have constructed two gene-disease associations networks (GDN), which are used to explore the shared genetic associations and disease comorbidity. Starting from the bipartite graph we generated biologically relevant network projections and constructed multi-relational gene-disease network in which nodes are diseases or genes, and edges indicate association between gene and disease. This bipartite graph consists of two disjoint sets of nodes, where one set corresponds to all known genetic disorders and the other set corresponds to all of our identified significant genes for SARS and HIV-1 infections. The list of disorders, disease genes, and associations between them were obtained from the Online Mendelian Inheritance in Man (OMIM) [58], a compendium of human disease genes and phenotypes (see details in the Methods section). We classified each disorder into one of 21 disorder categories based on the physiological system affected as introduced in Goh, Cusick, Valle, Childs, Vidal, Barabasi et al. [14].

**Figure 1:**
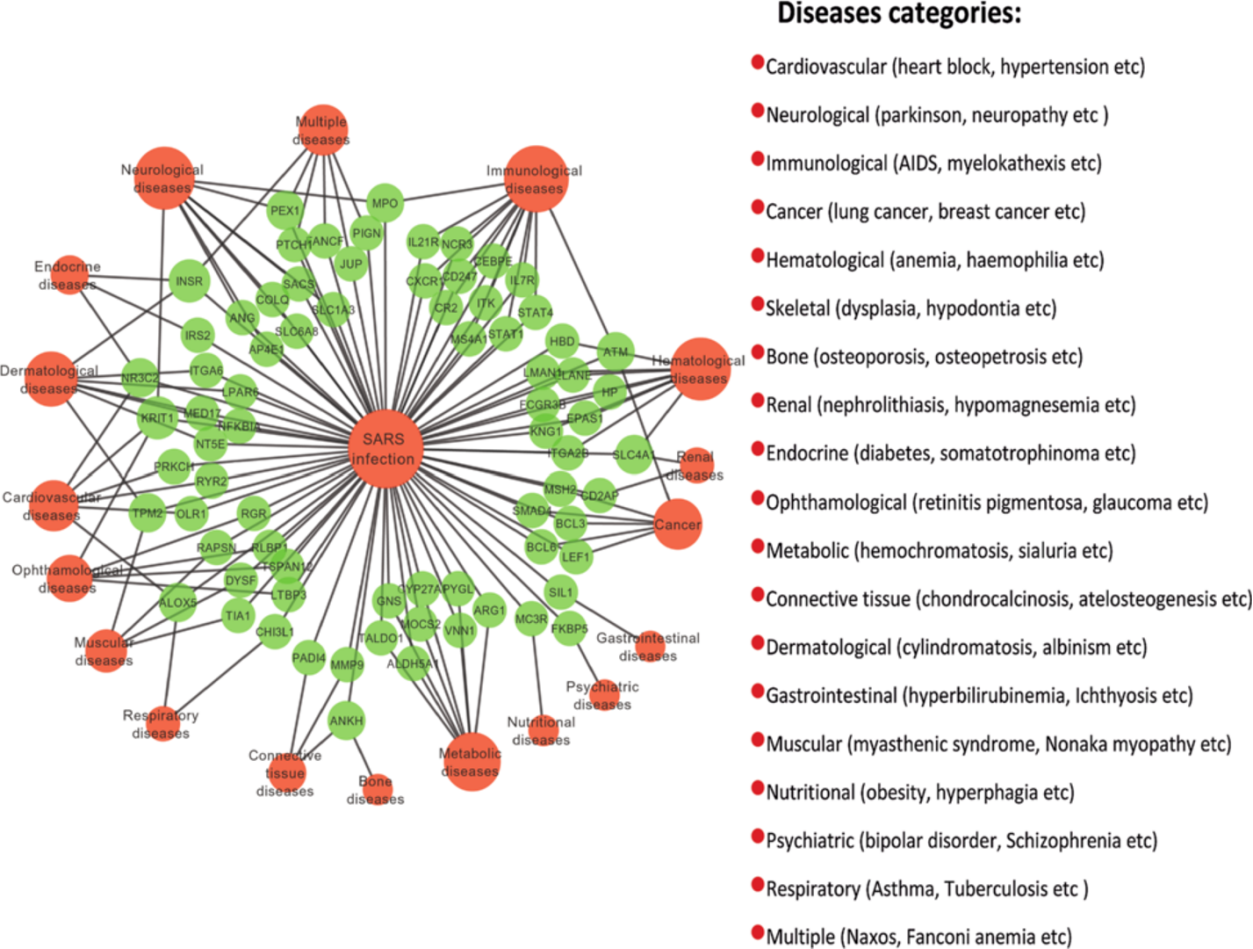
**The gene-disease association network centred on the SARS infection is constructed based on the different categories of diseases that are connected and showed comorbidities with the SARS infection through the different genes.** Red colour represents different categories of disorders and green colour represents different genes that are common with the other categories of disorders. The size of a disease node is proportional to the number of dysregulated genes shared between the infections/disorder groups. A link is placed between a disorder and a disease gene if mutations in that gene lead to the specific disorder.

**Figure 2:**
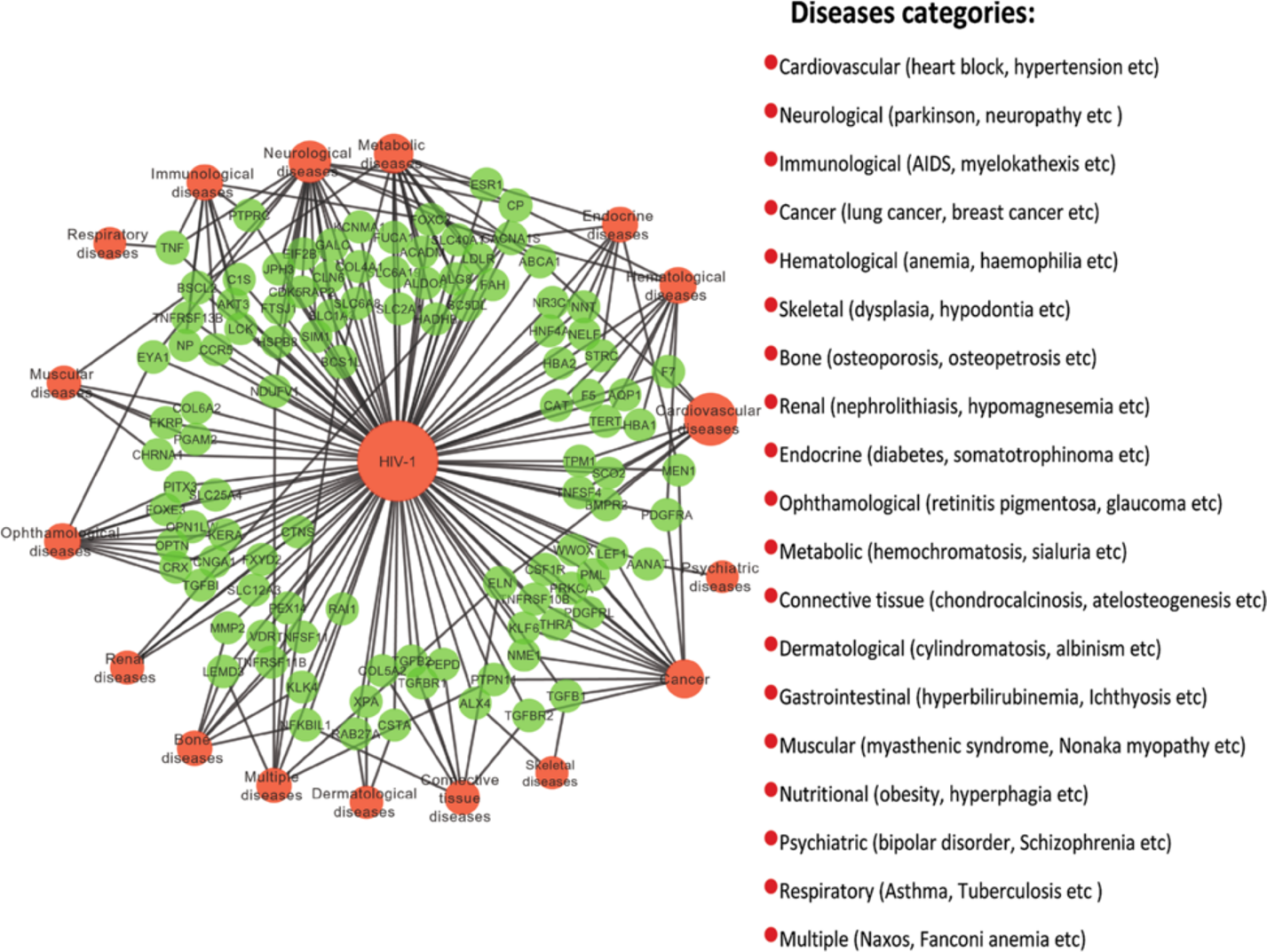
**The gene-disease association network centred on the HIV infection is constructed based on the different categories of diseases that are connected and showed comorbidities with the HIV-1 infection through the different genes.** Red colour represents different categories of disorders and green colour represents different genes that are common with the other categories of disorders. The size of a disease node is proportional to the number of dysregulated genes shared between the infections/disorder groups. A link is placed between a disorder and a disease gene if mutations in that gene lead to the specific disorder.

In the GDN, nodes represent diseases class or genes, and two disorders are connected to each other if they share at least one gene in which mutations are associated with both diseases groups (Figures 1 and 2). The number of interlinked genes between SARS infection and other diseases indicates that immunological, hematological, neurological, metabolic and dermatological diseases categories are strongly associated with the SARS infection (see Figure 1 and Additional file 3: Table S3). Few genes are also shared between more than 2 categories of diseases i.e those disease groups are also associated through at least that genes. For an instance, the gene ATM shared among SARS infection, cancer and immunological diseases. Therefore, cancer and immunological diseases are also interrelated through the gene ATM. Among all these disease classes immunological diseases class is tightly correlated with the SARS infection due to the highest number of genes (12 genes) shared between them. On the other hand, the number of associated genes between HIV infection and other diseases indicates that neurological, metabolic, cancer and hematological diseases categories are strongly correlated with the HIV infection (see Figure 2 and Additional file 4: Table S4). Few HIV dysregulated genes are also shared between more than 2 categories of diseases such as the gene TGFB1 is shared among HIV infection, cancer and skeletal diseases. It is notable that 11 significant genes (4 upregulated and 7 downregulated) are similarly dysregulated in the both SARS and HIV infections.

**Figure 3:**
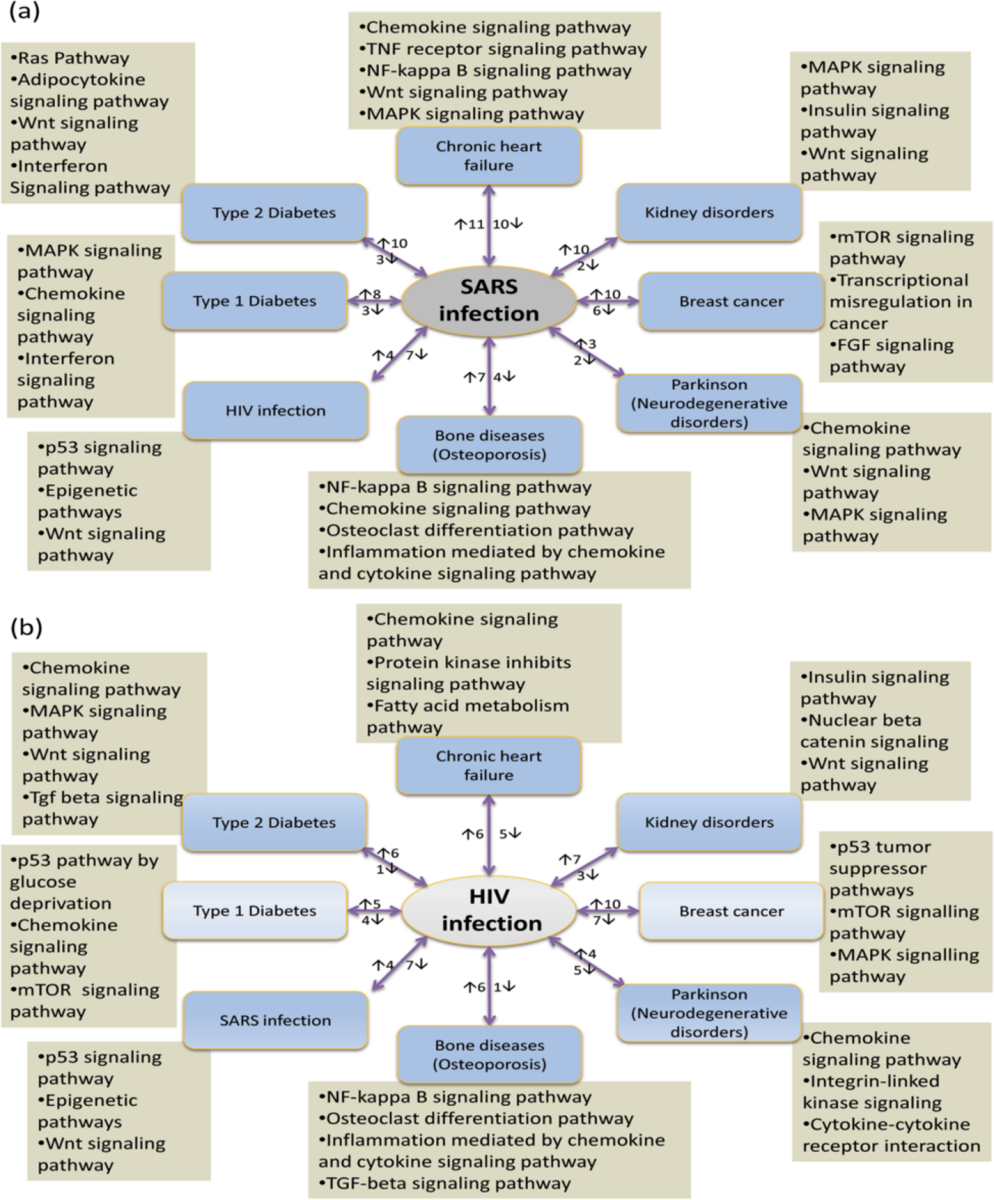
**Network of the eight diseases or infections (chronic heart failure, kidney disorders, breast cancer, parkinson, osteoporosis, HIV/SARS infection, type 1 and type 2 diabetes) that are associated and showed co-morbidities with the (a) SARS infection and (b) HIV infection through the shared genes and common pathways.** There are some highly up and down regulated genes that are common between SARS/HIV infection and the other 8 diseases or infections. Up and down arrows are indicated the common highly up and down dysregulated genes between SARS/HIV infection and the corresponding infection or disease.

To observe the association of SARS and HIV infections with other 7 important diseases (chronic heart failure, kidney disorders, breast cancer, parkinson, osteoporosis, type 1 and type 2 diabetes), we have collected mRNA microarray raw data associated with each disease from the Gene Expression Omnibus ( http://www.ncbi.nlm.nih.gov/geo/) accession numbers are GSE9006, GSE9128, GSE15072, GSE7158, GSE8977 and GSE7621 [59]. After several steps of statistical analysis we have selected the most significant over and under expressed genes for each infection and disease. We also performed cross compare analysis to find the common significant genes between each disease and SARS/ HIV-1 infection. We observed that SARS infection shares 21, 12, 16, 5, 11, 11, 11 and 13 genes corresponding to the chronic heart failure, kidney disorders, breast cancer, parkinson, osteoporosis, HIV-1 infection, type 1 and type 2 diabetes. On the other hand, HIV-1 infection shares 11, 10, 17, 9, 7, 11, 9 and 7 genes corresponding to the chronic heart failure, kidney disorders, breast cancer, parkinson, osteoporosis, SARS infection, type 1 and type 2 diabetes. Then we built disease–disease relationships network for SARS and HIV-1 infection with other diseases (see Figures 3 (a) and (b) and Additional file 5: Table S5 and Additional file 6: Table S6). Since genes do not function alone and they coordinate their activities in the form of complexes or molecular pathways. Therefore two diseases are potentially inter–correlated to each other if they share at least one commonly associated pathway. For this reason we have used reactome pathway database [60] and selected the pathways related to these 7 diseases as well as SARS and HIV-1 infections. We have observed that diseases and infections shared pathways between them as shown in Figures 3 (a) and (b) and Additional file 5: Table S5 and Additional file 6: Table S6.

**Figure 4:**
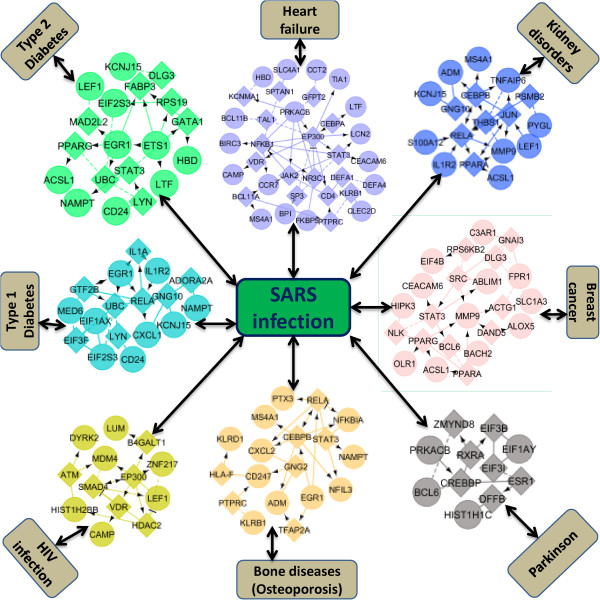
**Protein–protein interaction network of the eight diseases and infections (chronic heart failure, kidney disorders, breast cancer, parkinson, osteoporosis, HIV infection, type 1 and type 2 diabetes) that are associated and showed comorbidities with the SARS infection through the sharing protein subnetwork.**

**Figure 5:**
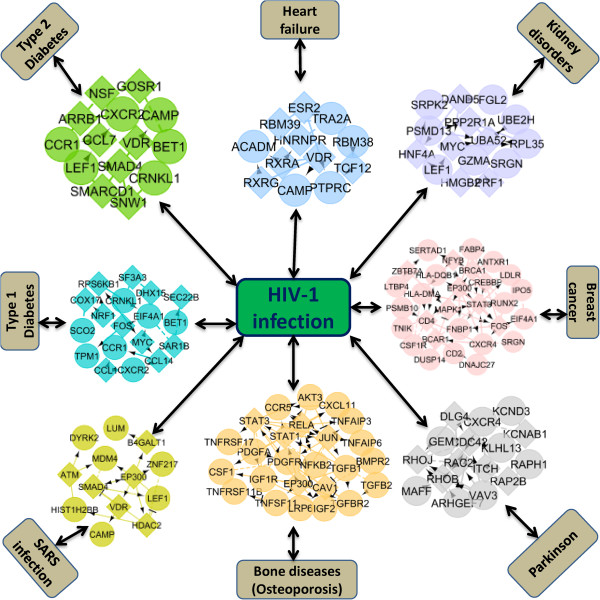
**Protein–protein interaction network of the eight diseases and infection (chronic heart failure, kidney disorders, breast cancer, parkinson, osteoporosis, SARS infection, type 1 and type 2 diabetes) that are associated and showed comorbidities with the HIV infection through the sharing protein subnetwork.**

Dysregulation in a protein subnetwork may yield the dysfunction of multiple protein subnetworks. Therefore, multiple diseases may be caused by the malfunction of a protein complex. So, two diseases are potentially related to each other if they share one or more commonly associated protein subnetwork. To identify the association between diseases based on the PPI subnetwork, we used significantly associated disease protein pairs data from the HPRD data base [61]. To find statistically significant associations among diseases, we built disease networks centred on the SARS and HIV infections in which two diseases are comorbid if there exists one or more protein subnetwork that are associated with both diseases. The disease similarity network and the protein-protein interaction network are integrated systematically and comprehensively in a simple and compact manner to formulate the disease comorbidity for the SARS and HIV-1 infections as shown in Figures 4 and 5. We showed that SARS and HIV infections shared PPI subnetworks with the other 7 diseases or infections similar to the gene-disease and pathway-disease associations as shown in Figures 4 and 5.

**Figure 6:**
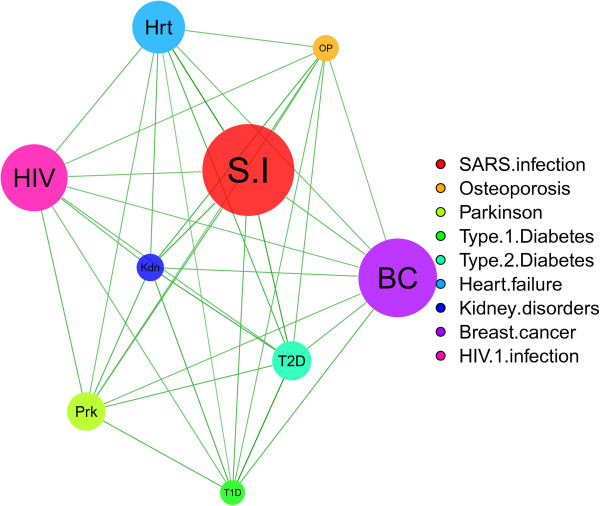
**Network of the comorbidities risk probability among 9 diseases and infections (SARS infection, Osteoporosis, Parkinson, Type 1 Diabetes, Type 2 Diabetes, Heart failure, Kidney disorders, Breast cancer and HIV-1 infection).** Comorbidities probability distances among infections and diseases are presented through the edges and variances are represented by the size of the nodes.

Based on the gene expression, protein-protein interaction and molecular pathways data, we have found that both SARS and HIV-1 infections have a strong association with other 8 diseases or infections (chronic heart failure, kidney disorders, breast cancer, parkinson, osteoporosis, HIV/SARS infection, type 1 and type 2 diabetes). These diseases and infections are also strongly correlated among them. We present the correlation strength and distance between a pair of these diseases and infections in Figure 6. We show that some diseases (such as kidney disorders, breast cancer, osteoporosis and heart failure) are more associated with the SARS infection (see Figure 6). Kidney disorder is also tightly connected with the HIV-1 infection. The probability of occurring comorbidities between the more tightly connected diseases is more than that of others.

**Figure 7:**
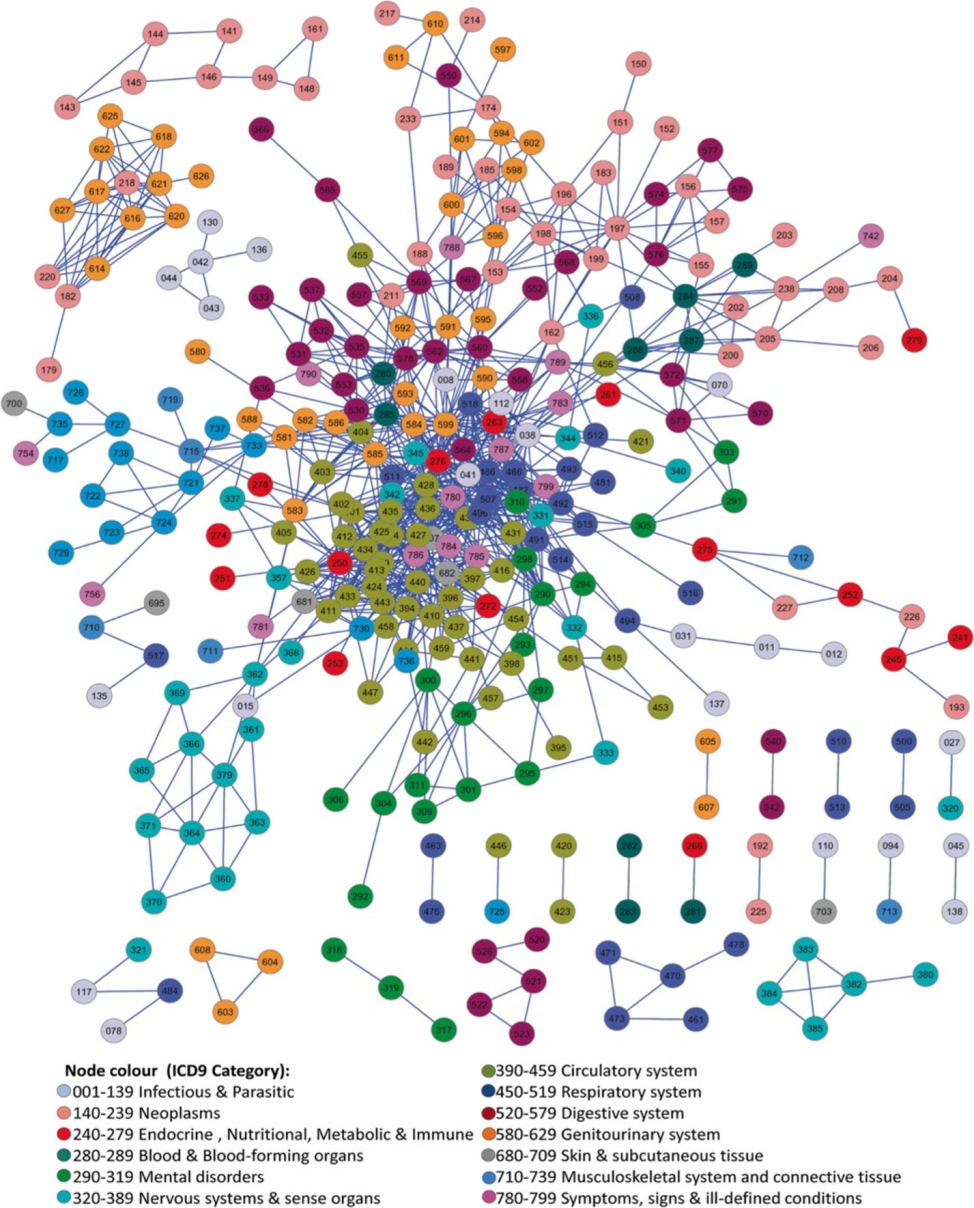
**Phenotypic Disease Networks (PDNs).** Nodes are diseases and links are correlations. Node colour identifies the diseases based on the ICD9 category. Only statistically significant links with *R* *R*_*ij*_ > = 20 and *ϕ* > = 0.06 are shown.

It is notable that the patient medical records contain important evidence regarding the co-occurrences of diseases affecting the same patient. So, we constructed a phenotypic disease comorbidity network using 32 million medical records of 13039018 patients data from MedPAR and analysed its structural properties to better understand the connections among diseases and infections. Nodes are unique diseases and edges indicate co-morbidity of the diseases. We included edges between disease pairs for which the co-occurrence is significantly greater than the random expectation based on population prevalence of the diseases. As pointed out in [2], the Relative Risk (*RR*_*ij*_) overestimates relations involving rare infections and diseases, and underestimates relationships between very common disorders or infections. On the other hand, *ϕ*-correlation underestimates comorbidity between rare and frequent diseases, and discriminates associations between disorders of similar appearances. Thus, we built a network by selecting only the statistically significant network edges having *RR*_*ij*_ ≥ 20 and *ϕ*_*ij*_ ≥ 0.06. Figure 7 summarises the set of all comorbidity associations among all diseases expressed in the study population by constructing a Phenotypic Disease Network (PDN). In the PDN, nodes are disease phenotypes identified by unique ICD-9-CM (The International Classification of Diseases) disease codes, and links connect phenotypes that show significant comorbidity according to the relative risk *RR*_*ij*_ ≥ 20 and the correlation *ϕ*_*ij*_ ≥ 0.06. Our phenotypic disease network consists of 336 unique diseases nodes and 1018 co-morbidity relationships.

**Figure 8:**
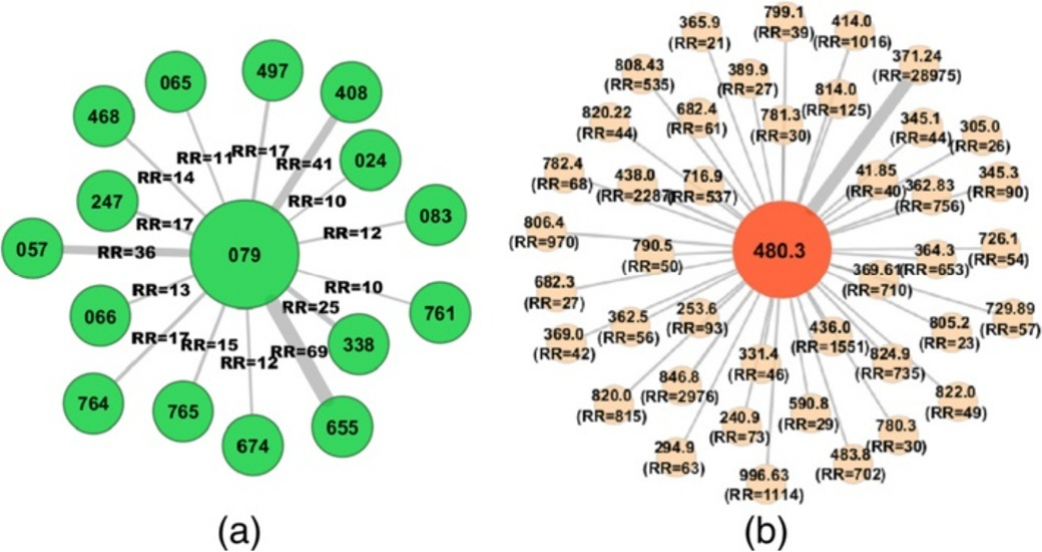
**Phenotypic Disease Networks (PDNs) for SARS infection.** Nodes are diseases and links are correlations. Node labels identify the ICD9 codes at the 3-digit category level in **(a)** and 5-digit category level in **(b)**. Only statistically significant links with relative risk *R* *R*_*ij*_ are shown.

SARS-associated coronavirus ICD-9-CM diagnosis code is 079.82, which is under the group of "Viral and chlamydial infection in conditions classified elsewhere and of unspecified site" and ICD-9-CM diagnosis code 079. Moreover, the ICD-9-CM code 480.3 is for the pneumonia due to SARS associated coronavirus. So we have considered both ICD-9-CM codes 079.82 and 480.3 for our phenotypic SARS comorbidity study. In our 3 digit code data we have considered 079 and for 5 digit code data we have considered 480.3. Considering the relative risk *RR*_*ij*_ ≥ 10 between the disease group 079 and other disorder categories, we have constructed the PDN as shown in Figure 8(a), and considering the relative risk *RR*_*ij*_ ≥ 20 between the disease group 480.3 and other disorder categories, we have constructed the PDN as shown in Figure 8(b). We presented only the most significant relative risk associations (see Additional file 7: Table S7 and Additional file 8: Table S8).

**Figure 9:**
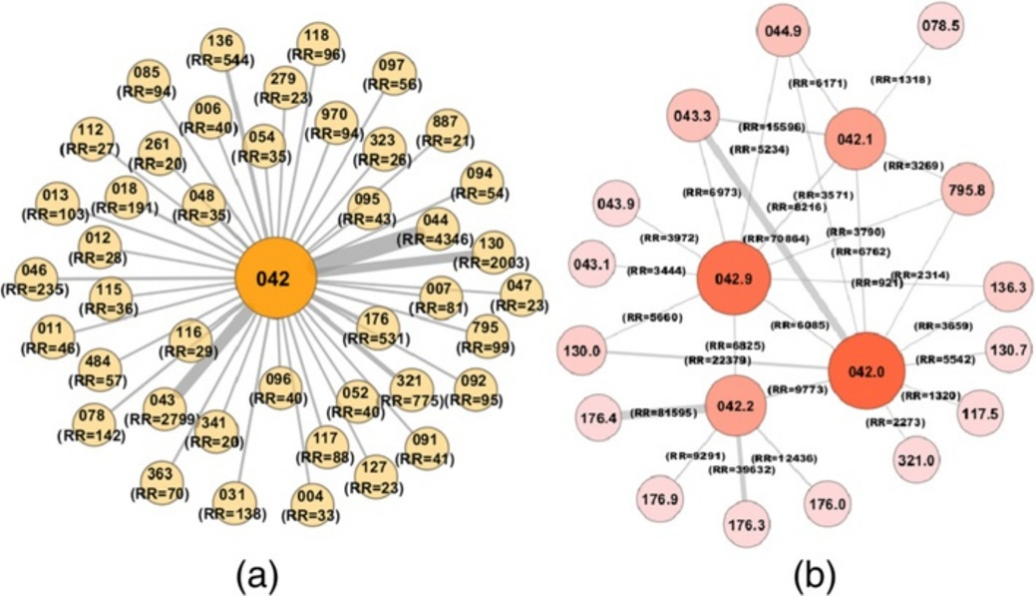
**Phenotypic Disease Networks (PDNs) for HIV-1 infection.** Nodes are diseases and links are correlations. Node labels identify the ICD9 codes at the 3-digit category level in **(a)** and 5-digit category level in **(b)**. Only statistically significant links with relative risk *R* *R*_*ij*_ are shown.

The ICD-9-CM diagnosis code for the Human immunodeficiency virus (HIV) infection is 042 to 044, which is under the group of "Infectious and parasitic diseases" and ICD-9-CM code (001–139). So we have considered both 3 digit and 5 digit ICD-9-CM codes for our phenotypic comorbidity studies related to HIV infection. Considering the relative risk *RR*_*ij*_ ≥ 20 between the disease group 042 and other disorder categories, we have constructed the PDN as shown in Figure 9(a) and considering the relative risk *RR*_*ij*_ ≥ 100 and *ϕ*-correlation *ϕ*_*ij*_ ≥ 0.06 between the disease groups under the sub categories of 042 and other disorder categories, we have constructed the PDN as shown in Figure 9(b). Only the most significant relative risk association is represented (see Additional file 9: Table S9 and Additional file 10: Table S10).

**Figure 10:**
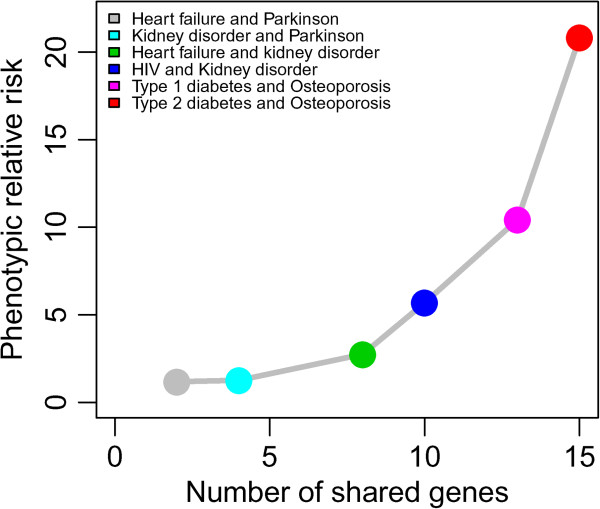
**Correlation between the number of shared genes and phenotypic relative risk for the disease comorbidities.**

To observe the trend of phenotypic relative risk corresponding to the number of shared genes between 2 diseases, we have computed the number of shared genes between two diseases and their corresponding phenotypic relative risk of the occurrence of comorbidities as shown in Figure 10. We observed that with increasing number of shared biomarker genes between 2 diseases, the phenotypic relative risk is also increased. We may predict existing diseases of a patient and the prospective disease comorbidities through the identification of highly up and down dysregulated genes. So based on the available data we could predict the disease comorbidities and the level of the comorbidities using the regression model as Figure 10.

**Figure 11:**
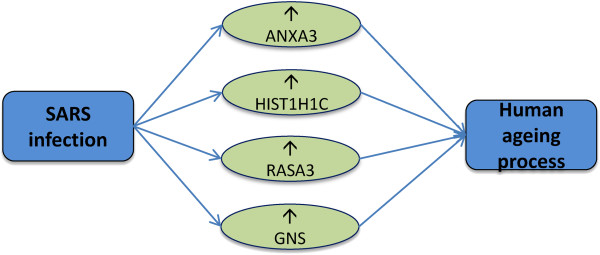
**Four genes (ANXA3, HIST1H1C, RASA3 and GNS) that are linked between SARS infection and ageing.** For the causes of these genes, ageing process of the SARS infected patients increase faster. Up arrows indicate the highly up dysregulated genes.

**Figure 12:**
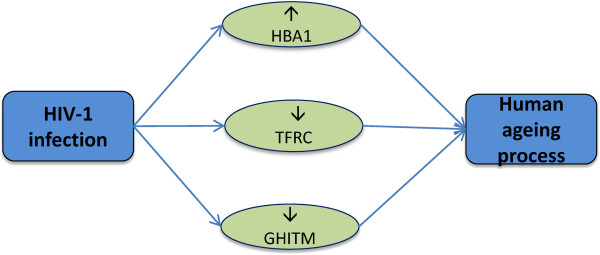
**Three genes (HBA1, TFRC and GHITM) that are linked between HIV-1 infection and ageing.** For the causes of these genes, ageing process of the HIV infected patients increase faster. Up and down arrows indicate the highly up and down dysregulated genes.

It is notable that ageing is also a "disease", not a natural process, for which age-related diseases increase exponentially with chronological time. So, to understand the impact of ageing on the disease comorbidities for SARS and HIV infections we have considered the ageing data from the GenAge database ( http://genomics.senescence.info/genes/human.html) [62, 63]. After cross comparing our 274 significant genes of SARS infection condition and 186 significant genes of HIV-1 infection condition with the 76 ageing related genes, it is observed that 4 genes (ANXA3, HIST1H1C, RASA3, GNS) are significantly over expressed in the both SARS infection and human ageing process as shown Figure 11, and 1 gene (HBA1) is significantly over expressed and 2 genes (TFRC, GHITM) are significantly under expressed in the both HIV infection and human ageing process as shown Figure 12. So from this observation it is recognised that SARS and HIV-1 infections are also linked with the ageing process of human through the regulation of distinct genes and pathways. On the other hand, ageing is directly linked with some other diseases and inflammation including cancers. Thus SARS and HIV infections also make comorbidities with other diseases through the genes related to ageing process. So the infection of SARS and HIV play multi way comorbidities with different diseases.

**Figure 13:**
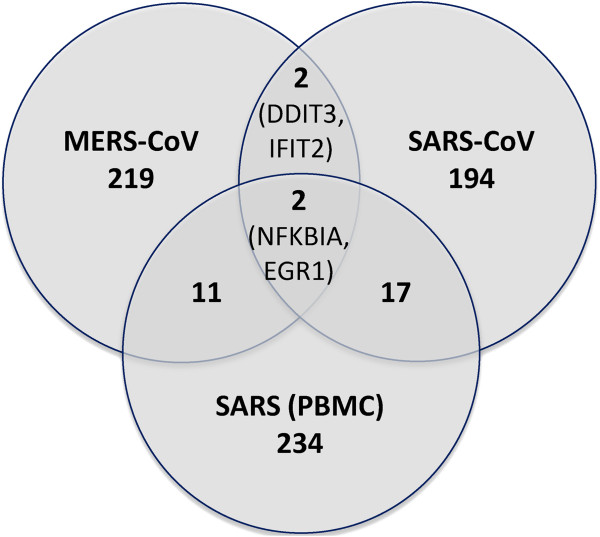
**Venn diagram of the highly over and under expressed genes for the SARS-CoV infection in lung and PBMC cells and MERS-CoV infection in the lung cells vs. corresponding to their Mock.**

Human lung epithelial cells are likely among the first targets to encounter invading severe acute respiratory syndrome-associated coronavirus (SARS-CoV) [32]. Thus, a comprehensive evaluation of the complex epithelial signalling to SARS-CoV is crucial to better understand SARS pathogenesis. Since both of the SARS-CoV and MERS-CoV infections cause severe lung pathology we compare and contrast the genes expression level of SARS-CoV infection and MERS-CoV infection. To compare between SARS-CoV and MERS-CoV infections, and the affect on the disease comorbidities, we have performed the time series microarray data analysis for the both types of infections on lung compared to controls. We have considered gene expression microarray data from the NCBI GEO ( http://www.ncbi.nlm.nih.gov/geo/query/acc.cgi?acc=GSE45042) [64]. From the analysis of SARS-CoV vs mock-infected controls (treated the same way except without the virus) we have found 215 genes are highly significant and from the analysis of MERS-CoV vs Mock we have found 234 gens are highly significant (see details in the Additional file 11: Table S11 and Additional file 12: Table S12). Interestingly, only 4 genes (NFKBIA, EGR1, DDIT31 and IFIT2) are common between these two infections (see Figure 13). However, only 2 genes (NFKBIA and EGR1) play an important role and differentially expressed among the both infections in lung and also in SARS infected PBMCs. Then from the hierarchical cluster analysis of the differentially expressed genes of the lung infection by SARS-CoV and MERS-CoV, we observed distinct groups of genes that were significantly changed over time (see Additional file 13: Figure S1 and Additional file 14: Figure S2, and Additional file 11: Table S11 and Additional file 12: Table S12).

**Figure 14:**
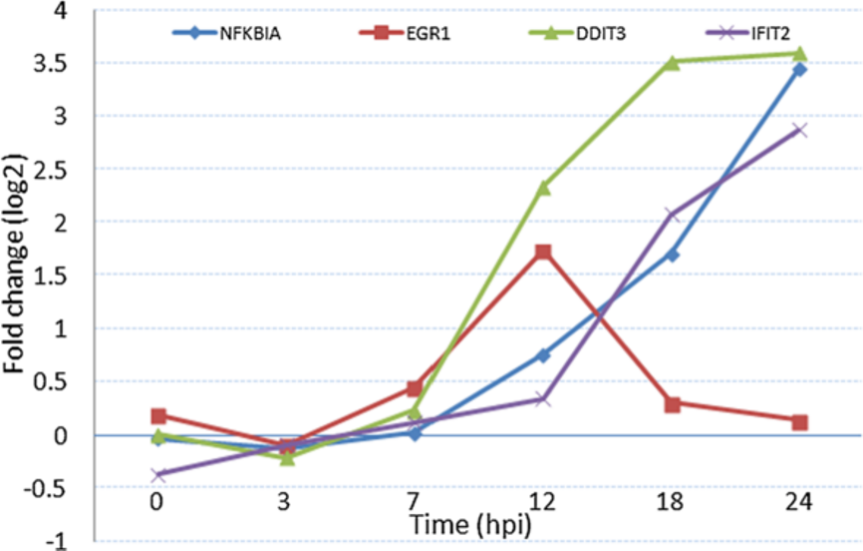
**Log fold changes of the expression level (y axis) of the MERS-CoV infected genes (x axis) corresponding to Mock in different time points.**

**Figure 15:**
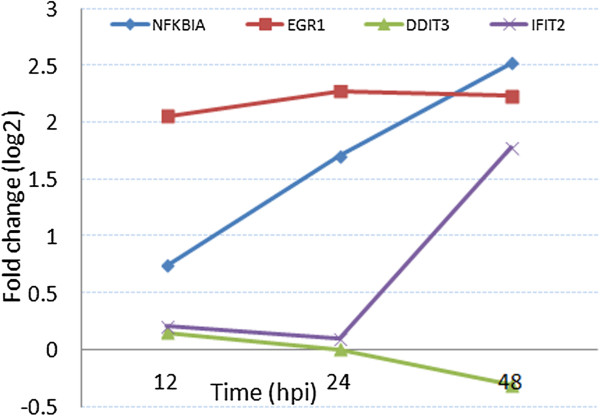
**Log fold changes of the expression level (y axis) of the SARS-Cov infected genes (x axis) corresponding to Mock in different time points.**

**Figure 16:**
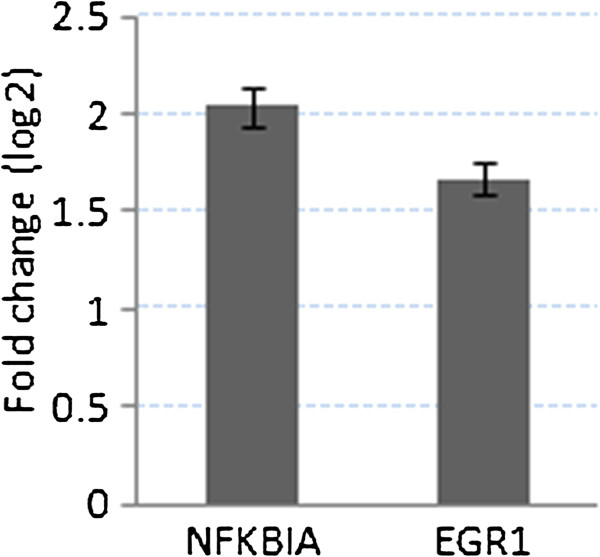
**Log fold changes of the expression level (y axis) of the SARS infected NFKBIA and EGR1 genes (x axis) corresponding to Mock.**

The log fold changes of the common 4 genes (NFKBIA, EGR1, DDIT31 and IFIT2) expression level for the infection of MERS-CoV and SARS-CoV are presented in the Figures 14 and 15. We observed that the log fold changes of NFKBIA genes expression level is sharply upregulated in the both types of infections corresponding to time point. So NFKBIA is an important bio-marker for the both MERS-CoV and SARS-CoV infections. It is also observed that the inflammatory genes NFKBIA is a highly over expressed in the both PBMCs and lung cells for the infection of SARS and also for the infection of MERS in the lung cells (see Figure 16). Indeed, the immune system plays a pivotal role in the outbreak of the inflammatory state. So in case of SARS infection, the NFKBIA gene plays an important role for the disease comorbidities.

On the other hand, similar diseases share common genes and could be treated by the same drugs [17], which may allow us to make predictions for new uses of existing drugs. For an instance, the anti-diabetic drug metformin plays a major protective effect against cancer development and increases significantly higher survival rate of the cancer patients [65]. The finding is that the earlier the metformin regimen was initiated, the greater the preventive benefit for the cancer patient. There is an evidence that the antiviral medication, ribavirin, does not work in case of SARS infection [66]. To this end, we used Connectivity Map (Cmap), which is a database of more than 1,400 drug transcriptional signatures in several cell lines [67]. This database allows to identify of molecules that induce similar or opposite transcriptional changes relative to the query signature, based on their connectivity enrichment scores. As a query signature we used our 274 highly dysregulated genes for the SARS infection. We generated the connectivity score value ranges between +1 and -1, where a highly positive score indicates that the drug induces changes similar to those induced by viral infection, while a highly negative score indicates that the drug reverses the expression of the SARS signature. Based on the connectivity score we have selected most potential positive and negative regulators of SARS viral response (see details in the Additional file 15: Table S13). Potential negative regulators indicate that drugs reverse the SARS signature gene expression. Among the negative potential regulator, the drug molecule tetracycline, zalcitabine, gibberellic acid, prestwick-642 and sulfaquinoxaline are more potential for the MCF7 cell line and vorinostat for the HL60 cell line. Based on the data demonstrate the efficacy of different drug against SARS virus can be predicted effective drug treatment for the emergent viruses. Furthermore, immunomodulatory drugs that reduce the excessive host inflammatory response to respiratory viruses have therapeutic benefit to reduce the SARS infection as well as disease comorbidities.

### Discussion

We presented and analysed multi-relational disease comorbidity relationships of SARS and HIV-1 infections with other diseases or infections based on the associations of genetics, proteomics, molecular signalling pathways and phenotypic disorders. The combination of molecular biology, genetics and clinical medicine has greatly facilitated understanding of how different diseases relate to each other. Based on the combined genetics, PPIs, pathways and clinical data, our disease networks can disclose potentially novel disease relationships that have not been captured by previous individual studies. The underlying hypothesis behind this line of research is that once we catalogue all disease-related genes, PPI complex and signalling pathways, if we do not consider environmental changes, we will be able to predict the susceptibility of each individual to future diseases using various molecular biomarkers and it will help us to enter an era of predictive medicine. Our results indicate that such a combination of molecular and population-level data could help to build novel hypotheses about disease mechanisms. Furthermore, if two or more diseases have associated comorbidity, the occurrence of one of them in a patient may increase the likelihood of developing the other diseases.

We have also studied the differences between MERS-CoV and SARS-CoV in the host response. This enables rapid assessment of viral properties and the ability to anticipate possible differences in human clinical responses to MERS-CoV and SARS-CoV and their impact on comorbidities with respect to the general comorbidities conditions. We used this information to predict potential effective drugs against SARS-CoV, a method that could be more generally used to identify candidate therapeutics in future disease outbreaks. These investigation approach may also help to generate hypotheses and make rapid advancements in characterising the new viruses.

We also found that patients’ response of SARS appears to be mainly an innate inflammatory response using NFKBIA, rather than any specific immune response against a viral infection such as HIV. However, HIV infection and highly active antiretroviral therapy (HAART) also increase the immune reconstitution inflammatory syndrome (IRIS) and inflammation through the NF- *κ*B pathways [68]. Moreover we have studied before about the impact of HIV infection on bone diseases and infection (e. g. osteoporosis and osteomyelitis). We observed that genes (e.g. RANKL) and pathways (e.g. NF- *κ*B) that are dysregulated by HIV infection also impact on the bone remodelling and bone related diseases. It is also recognised that inflammation plays a role in cancer aetiology, and various studies have found that inflammation may causes IRIS, obesity and tumour-promoting effects [69]. Moreover, inflammation is an important concomitant cause of many major age-associated pathologies such as cancer, neurodegeneration and diabetes [70]. Our study provides important evidence to associate diseases with the ageing process at the system level and helps to understand more about the comorbidities of the complex diseases. The ageing process itself is accompanied by a chronic low-grade inflammation, which is termed "inflammageing". The combination of metabolic-driven and age-driven inflammatory pathways plays a pivotal role in disease progression. This observation suggests that inflammageing and meta-inflammation can share stimuli and pathogenic mechanisms for comorbidities.

We suppose that what is happening for the comorbidities we investigate is similar to what found for prions [71, 72]. Similar to most infectious agents, prion causes inflammatory responses by activating innate immunity through glial cells in the brain.

**Figure 17:**
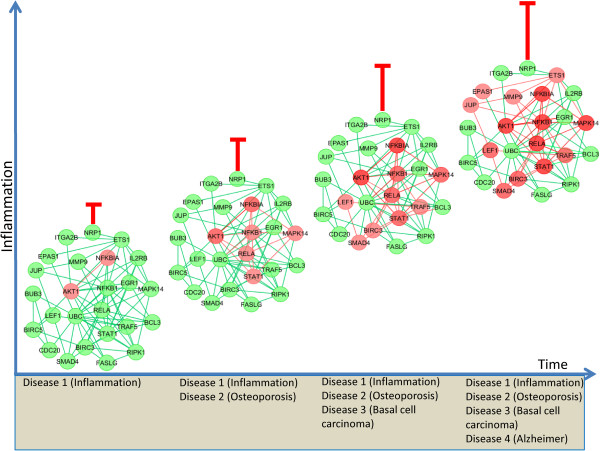
**Progressive temporal activation of pathways.** A schematic view of networks becoming disease comorbidities increased for the perturbation of the pathways dysregulation advances with time. The red circles indicate increased levels of dysregulated gene expression relative to control and the red linked indicate dysregulated pathways that have been increased from infection as compared with normal control. The green indicated transcripts that are the same in control and infection condition. The four panels represent the network with time intervals of the infection progression. With time the inflammation confidence level is increased which is indicated by confidence interval.

The complete transcriptome of the prion brain at 10 different time points is observed during the 22-week period [71, 72]. At the beginning of the disease, both normal and diseased mouse networks were the same. Although the disease started in the most unique network of prion accumulation and replication it is progressed to the other networks. Based on this approach we may propose a pathway model for comorbidities how hubs genes dysregulate several other pathways to influence comorbidities. The number of dysregulated pathways could be proportional to the amount of dysregulation of hub genes. Our pathway model may states that the hubs that are over turned on, may direct the signal to the different pathways creating comorbidities as shown in Figure 17. For the infection, one of the pathways related to the inflammation starts dysregulation. With increasing time, both confidence level of inflammation and the number of dysregulated pathways are increased. Moreover, with the increasing of inflammation the number of diseases for the comorbidities may increase. So initially infection dysregulates one signalling pathway of any cells and that causes other pathways may be dysrupted. In this way disrupting pathways increase more diseases in the same patient and make multimorbidity.

Disease genes play a central role in the human interactomes. Overlapping component genes serve as bridges across the relatively independent functional modules or pathways. So perturbation in one pathway, such as the NF- *κ*B signalling pathway, could be propagated throughout the other relevant pathways. We found SARS and HIV-1 infections share 11 significantly dysregulated genes as well as molecular pathways. Both SARS and HIV-1 viruses may infect and find an already existing comorbidity or generate a new comorbidity through the perturbation of the infected pathways. Furthermore, it may provide us an opportunity to investigate the role of other genes from the same pathway in the disease space. Therefore, pathways could be used to represent the underlying biology of diseases and make prediction of disease comorbidities. In most of the cases, the correlativeness among genes, pathways and diseases are many-to-many, e.g. a disease is associated to many different genes and pathways; and a pathway is associated to many different diseases. This study suggests that a single pathway can be involved in many diseases whereas a disease may have dysregulation in many biological processes. Hence, if a drug is already available to treat a disease through modulating the activity of a pathway, then it could potentially be used to treat other diseases that are strongly linked with the same pathway. On the other hand, when a disease shows dysregulation in multiple pathways, a pathway-guided combined drug may be employed in the treatment. Moreover, the protein subnetwork–based approach to diseases may aid in drug discovery, in fact it can potentially be used to treat other diseases that are linked to the same protein complex. Thus, our findings not only potentially help us to understand how different diseases are related based on their underlying molecular mechanisms but also provide insights into the design of novel, protein complex-guided therapeutic interventions for diseases.

#### Personalised medicine: guidelines for predicting comorbidities

Extending the concept of subclassifying patient cohorts to the single patient level refers to as personalised medicine. During the last few years, acceptance level of the personalised medicine is sharply increased as it has been apparent that standard treatment approaches are rarely efficient across the entire patient population. Advances in high-throughput molecular assay technologies in the fields of genomics, proteomics and other "omics" is increasing the diagnostic and therapeutic strategies for personalised treatment. As a result, declining per-sample cost has given rise to numerous public repositories of biomolecular data. In particular, the availability of these data sets for many different diseases presents a ripe opportunity to use data-driven approaches to advance our current knowledge of disease relationships in a systematic way. The identified disease patterns can then be further investigated with regards to their diagnostic utility or help in predicting novel therapeutic targets. Medicine will focus on each individual patient. It will become intrinsically proactive and will increasingly focus on wellness rather than disease. Proactive and personalised medicine will bring fundamental changes to healthcare, taking carefully targeted preventative or therapeutic action at the earliest indications of risk or disease comorbidities.

We are entering into the genomic era of medicine, where a patient’s genetic/genomic data is becoming important for clinical decision making, including disease risk assessment, disease diagnosis and subtyping, drug therapy and dose selection, risk assessment for adverse drug reaction, and family planning [73]. Today multi-scale and complex biomedical data are gathered and analysed to uncover combinations of predictive disease profiles. Our genome, as well as multiple proteomes, multiple transcriptomes, multiple metabolomes, and other personalised data sets obtained at different points in our lives, will be readily available at affordable prices for each individual. In the near future, clinicians will have to consider genetic/genomic implications to patient care throughout their clinical workflow, including electronic prescribing of medications. Therefore, for the implementation of the personalised medicine system, a model could be developed that will take individual genetic data. Dysregulated biomarker genes will be identified from this genetic data and disease will be identified from the gene–disease association database. Based on the information of the existing disease, the model will predict disease comorbidities using the disease–disease associations database. This will provide us to detect many diseases at the earliest detectable phase, even weeks, months, or maybe years before the symptoms appear and it will afford crucial insights into optimizing of our wellness. Thus, personalised medicine will give fundamental new insights into disease mechanisms, and hence will open new opportunities for diagnosis, therapy and prevention from the disease comorbidities.

## Conclusion

In this study, we have considered all available categories of omics and phenotypic data to quantify the SARS and HIV-1 infections centred comorbidity associations. We have shown that the phenotype disease network (PDN) has a heterogeneous structure where some diseases are highly connected while others are hardly connected at all. Our findings showed that disease progression can be represented and studied using network methods, offering the potential to enhance our understanding of the origin and evolution of human diseases. Detecting comorbidity in a large population is of clinical interest due to the fact that it may reveal new information useful for cause of diseases as well as for new treatment strategies. This study demonstrates the value of an integrated approach in revealing disease relationships and new opportunities for therapeutic applications. So we can say that this kind of approach will be helpful for making evidence-based recommendations about disease comorbidities. Moreover, considering environmental factors (such as physiological stress, diet), ethnic group and gender discriminations are important factors in the comorbidity analysis. Our network approach could be extended as a comorbidity map by integrating diet, exercise and other factors as in [74].

## Methods

### Data

The gene-disease associations data used in this study were collected from the Online Mendelian Inheritance in Man (OMIM) database ( http://www.ncbi.nlm.nih.gov/omim/). This OMIM database is the best-curated repository of all known disease genes and their associated disorders [75, 76]. Genotype-phenotype relationships, as summarised in the OMIM database, contained more than 5000 human disease-genes associations involving 1500 diseases and 3000 disease associated genes. Each entry of the OMIM is composed of four fields, the name of the disorder, the associated gene symbols, its corresponding OMIM id, and the chromosomal location. We selected the entries with the "(3)" tag, for which there is strong evidence that at least one mutation is cause of the disorder. OMIM initially focused on monogenic disorders but in recent years has expanded to include complex traits and the associated genetic mutations that confer susceptibility to these common disorders [58]. Subsequently we classified each disorder into 21 primary disorder classes based on the physiological system affected as introduced in Goh, Cusick, Valle, Childs, Vidal and Barabasi et al. [14]. Disorders having distinct multiple clinical features are assigned to the "multiple" class. This classification scheme reflects the phenotypic similarities among diseases in the same class and has been successfully used in the recent studies of systematic disease analysis [77].

The gene expression data used in this study was obtained from the NCBI Gene Expression Omnibus (GEO) ( http://www.ncbi.nlm.nih.gov/geo/) [59]. We have considered 10 different data sets for our analysis (accession numbers are GSE1739, GSE45042, GSE17400, GSE9006, GSE9128, GSE15072, GSE7158, GSE8977, GSE18464 and GSE7621) [32, 55, 57, 64, 78–82]. These data sets contain data from the patients of different age and sex. After several rounds of filtering, normalization and statistical analysis, we had microarrays representing SARS, MERS, HIV-1 infections and 7 other human diseases (heart failure, kidney disorders, breast cancer, parkinson, osteoporosis, type 1 and type 2 diabetes).

The protein-protein interaction (PPI) data for human was obtained from the Human Protein Reference Database (HPRD) [61]. HPRD contains the maximum number of PPI data among all publicly available literature-derived databases for human PPI [83]. We have used the reactome knowledge base of human biological pathways database for our pathways association analysis [60]. For the cross compare analysis between the SARS and HIV infections, and ageing process we have download ageing data from the human ageing genomic resources ( http://genomics.senescence.info/download.html) [62, 63]. They have collected human ageing genes after an extensive review of the literature. These genes are commonly dysregulated during the ageing process.

To test the validity of the proposed disease associations, we examined the disease co-occurrence information at the population level. We obtained statistically significant pairwise comorbidity associations reconstructed from over 32 million medical records in the US Medicare claims database recorded in the ICD-9-CM format ( http://www.icd9data.com), which are frequently used for epidemiological and demographic studies and collected from [2]. We used MedPAR records from 1990 to 1993, where the dates and reasons for all hospitalizations were reported in ICD-9-CM format and it contains the diagnoses of 13039018 elderly patients. Each record consists of the date of visit, a primary diagnosis and up to 9 secondary diagnosis. All diagnoses are specified by ICD9 codes of up to 5 digits. The first three digits specify the main disease category while the last two are used to give additional information about the disease. In total, the ICD-9-CM classification consists of 657 different categories at the 3 digit level and 16,459 categories at the 5 digit level [2].

To determine whether some existing drug compounds can reverse the SARS infection signature, we used the publicly available Connectivity Map (Cmap) database [67]. Cmap provides associations among genes, chemicals and disease or infection conditions. It is a collection of genome-wide transcriptional data from cultured human cells treated with 1,400 different compounds.

### Methods

The method of global gene expression analysis using oligonucleotide microarrays has proven to be a sensitive method to develop and refine the molecular determinants of human disorders [55]. Using this technology, we compared the gene expression profiles of SARS, HIV and other diseases. To avoid the problems of comparing microarray data of different platforms and experimental systems, we normalized the gene expression data in each microarray sample (disease state or control) using the Z-score transformation (*Z*_*ij*_) for each disease gene expression matrix using , where *SD* is the standard deviation, *g*_*ij*_ represents the expression value of gene *i* in sample *j*. This transformation allows for the direct comparison of gene expression values across various microarray samples and diseases. To combined more than one data series or experiments for a given disease, we employed a linear regression approach to obtain a combined t-test statistic between two conditions. Data were *log*_2_-transformed and we calculated expression level for each gene using a linear regression model : *Y*_*i*_ = *β*_0_ + *β*_1_*X*_*i*_, where *Y*_*i*_ is the gene expression value and *X*_*i*_ is a disease state (disease or control). The coefficients *β*_0_ and *β*_1_ are the parameters of this model and were estimated by least squares. The t-test statistic, when estimating the value of *β*_1_, is the same as the standard t-test statistic between disease and control states.

Time series microarray gene expression data analysis was divide into two steps: pre-processing and identification of statistically significant points by t-test, ANOVA and regression analysis to find differently expressed gene profiles in different time points. In the first step, we pre-processed the experimental data using different statistical methods and finally followed by post less normalization, recommended by the Golden Spike Project [84]. In the second step, we have used a most suitable method "maSigPro" (microarray Significant Profiles) to identify differentially expressed genes in time-course microarray experiments, which is a two step regression method successfully applied on more than one groups of time-series [85, 86]. This two steps regression strategy is used to find genes with significant temporal expression changes and significant differences between experimental groups. This procedure first adjusts this global model by the least-squared technique to identify differentially expressed genes and selects significant genes applying false discovery rate control procedures. Then stepwise regression is applied as a variable selection strategy to study differences between experimental groups and statistically significant profiles. After finding differentially gene expression profiles among the group of experiments, the next step is to cluster them according to their profile similarities. The hierarchical clustering and the median gene expression profiles of clusters are performed according to the "maSigPro" package in R [85].

Student’s unpaired T-test was performed to identify genes that were differentially expressed in patients over normal samples and significant genes were selected. A threshold of at least 1.5 fold change and a *p* value for the t-tests of less than 0.01 were chosen. In addition, a two-way ANOVA with Bonferroni’s post-hoc test was used to establish statistical significance between groups (< 0.01). Pathways and functional categories were considered as over-represented when Fisher’s exact test *p* value was < 0.01. For presenting the signalling and interaction pathways of the different significant genes, we used cytoscape for data integration and network visualization [87, 88] and reactome functional interaction (FI) cytoscape plugin for knowledge base of human biological pathways and network processes [60].

For the gene disease association, we have considered the neighbourhood based benchmark and topological methods, which are better suited to our networks [89]. In this case, topological refers to methods that rely only on the structure of the network to draw conclusions. We construct a GDN from gene–disease associations where the node in the network can be either a disease or a gene. This network can also be regarded as a bipartite graph. Diseases are connected when the diseases share at least one significant dysregulated genes. Let a particular set of human diseases *D* and a set of human genes *G*, gene-disease associations attempt to find whether gene *g* ∈ *G* is associated with disease *d* ∈ *D*. If *G*_*i*_ and *G*_*j*_, the sets of significant up and down dysregulated genes associated with diseases *i* and *j* respectively, then the number of shared dysregulated genes  associated with both diseases *i* and *j* is as follows:
1

The co-occurrence refers to the number of shared genes in the GDN. The common neighbours is the based on the Jaccard Coefficient method, where the edge prediction score for the node pair is as:
2

where *E* is the set of all edges. The number of shared pathways and protein subnetwork that links between diseases *i* and *j* are calculated using the equation 1 and the link prediction score is measured using the equation 2.

To estimate the correlation starting from disease co-occurrence, we need to quantify the strength of disease association for comorbidities by dipicting a distance between two diseases. For the analysis of the phenotypic data, we used the Relative Risk (*RR*_*ij*_) as the quantified measures of comorbidity tendency of two disease pairs and checked *ϕ*-correlation (*ϕ*_*ij*_) to measure the robustness of the comorbidity associations. The *RR*_*ij*_ is observing in a pair of diseases *i* and *j* affecting the same patient. When two diseases co-occur more frequently than expected by chance, we will get *RR*_*ij*_ > 1 and *ϕ*_*ij*_ > 0. However, *RR*_*ij*_ and *ϕ*_*ij*_ are not independent of each other and each carries unique biases that are complementary [1, 2]. So, we used both measures of comorbidity to ensure the robustness of our investigations. The *RR*_*ij*_ allows us to quantify the co-occurrence of disease pairs compared with the random expectation which is calculated as:
3

where *N* is the total number of patients in the population, *P*_*i*_ is the incidences/prevalences of disease *i*, *P*_*j*_ is the incidence of disease *j* and *C*_*ij*_ is the number of patients that have been diagnosed with both diseases *i* and *j*. For *RR*_*ij*_ > = 1 comorbidity is larger than expected by chance and for *RR*_*ij*_ < 1 comorbidity is smaller than expected by chance. To calculate the significance of the relative risk *RR*_*ij*_, we used the Katz, Baptista, Azen and Pike et al. method to estimate confidence intervals [90]. According to their estimation, the 99% confidence interval for the *RR*_*ij*_ between two diseases *i* and *j* is calculated by: Lower bounds of the confidence interval (*LB*) = *RR*_*ij*_ ∗ *exp*(-2.56 ∗ *σ*_*ij*_) and Upper bounds of the confidence interval (*UB*) = *RR*_*ij*_ ∗ *exp*(2.56 ∗ *σ*_*ij*_), where *σ*_*ij*_ is given by: . Disease pairs within the 99% confidence interval are only considered if the *LB* value is larger than 1 when *RR*_*ij*_ is larger than 1, or if the *UB* value is smaller than 1 when *RR*_*ij*_ is smaller than 1.

Relative risk measure is intrinsically biased towards overestimation of relationships between rare diseases and underestimates the co-morbidity of more frequent diseases [2]. This bias can be reduced by introduction of a *ϕ*-correlation measure. We can quantify the strength of comorbidities by calculating the correlation coefficient associated with a pair of diseases *i* and *j* as:
4

where *C*_*ij*_ is the number of patients affected by both diseases, *N* is the total number of patients in the studied population, and *P*_*i*_ and *P*_*j*_ are the morbidity or incidence of the *i*^*th*^ and *j*^*th*^ diseases respectively. The *ϕ*-correlation is the Pearson’s correlation for the variables which only take 0 or 1 values [91]. For *ϕ*_*ij*_ > 0 comorbidity is larger than expected by chance and for *ϕ*_*ij*_ < 0 comorbidity is smaller than expected by chance. We can determine the significance of *ϕ* ≠ 0 by performing a *t*-test. This consists of calculating *t* according to the formula: , where *n* is the number of observations used to calculate *ϕ*.

To predict the comorbidities considering the primary or index disease we have calculated the conditional relative risk (conditional *RR*_*ij*_) as follows:
5

for all possible disease pairs *i* and *j*, for the cases that one index disease (*I*) is present (*k* = *true*) or absent (*k* = *false*). Then for each pair of diseases, we say that *i* and *j* are mediated by that index disease if the  is significantly different (higher or lower) from  (*p* = 0.01).

We have weighted the edges using a mutual information metric which quantifies how much greater the edge relationship is with respect to co-occurrence. The mutual information weight between two diseases *i* and *j* is defined as
6

where *C*_*ij*_ is the observed co-occurrence and *P*_*i*_ and *P*_*j*_ are the morbidity or prevalence of the *i*^*th*^ and *j*^*th*^ diseases respectively.

To compare between SARS-CoV and MERS-CoV, a gene set enrichment analysis was undertaken using GSEA [92]. To find out the correlation (similarities) and distance (dissimilarities) among the diseases from the integrated analysis of multidimensional data (gene expression and protein protein interaction), we have applied Euclidian distance measurement and metric multi-dimensional scaling (MDS) using majorization [93]. MDS is a set of methods for discovering hidden structures in multidimensional data. Based on a proximity matrix derived from variables measured on objects as input entity, these distances are mapped on a lower dimensional spatial representation. Optimization problem is used to find mapping in target dimension of the data based on given pairwise proximity information while minimize the objective function. The particular objective function (or loss function) we used in this work is a sum of squares, commonly called stress. We used majorization to minimize stress and this MDS solving strategy is known as SMACOF (Scaling by MAjorizing a COmplicated Function). Stress majorization is an optimization strategy used in multidimensional scaling (MDS) where, for a set of *nm*-dimensional data items, a configuration *X* of n points in *r*(<< *m*)-dimensional space is sought that minimizes the stress function *σ*(*X*). Here r is 2 that means the (*r* × *n*) matrix *X* lists points in 2-dimensional Euclidean space. We have applied the cost function *σ* to measures the squared differences between ideal (m-dimensional) distances and actual distances in r-dimensional space as follows:
7

*X*_1_ of dimension *n*_1_ × *p* as the individual’s or judge’s configuration, and *X*_2_ of dimension *n*_2_×*p* as the object’s configuration matrix. The least squares metric multidimensional scaling or MDS problem is the minimization of *σ* and over all *m*×*p* configurations *X*. Here *w*_*ij*_ are given non-negative weights and *d*_*ij*_ are given non-negative dissimilarities. The *d*_*ij*_(*X*) are the Euclidean distances between rows *i* and *j* of *X*. Thus
8

where *w*_*ij*_ ≥ 0 is a weight for the measurement between a pair of points (*i*,*j*), *d*_*ij*_(*X*) is the Euclidean distance between *i* and *j*, and *δ*_*ij*_ is the ideal distance between the points (their separation) in the *m*-dimensional data space. Note that *w*_*ij*_ is used to specify a degree of confidence in the similarity between points (e.g. 0 can be specified if there is no information for a particular pair). A configuration *X* which minimizes *σ*(*X*) gives a plot in which points that are close together correspond to points that are also close together in the original *m*-dimensional data space. Programming scripts are freely available at http://www.cl.cam.ac.uk/~mam211/comoR/.

## Electronic supplementary material

Additional file 1: **Table S1.** Highly statistical significantly differential expressed genes between SARS and control group in PBMCs. (XLSX 26 KB)

Additional file 2: **Table S2.** Highly statistical significantly differential expressed genes between HIV and control group. (XLSX 21 KB)

Additional file 3: **Table S3.** The gene-disease association related to the SARS infection with the different categories of diseases. (CSV 13 KB)

Additional file 4: **Table S4.** The gene-disease association related to the SARS infection with the different categories of diseases. (CSV 15 KB)

Additional file 5: **Table S5.** Pathways related to the 274 genes that are highly over and under expressed for the SARS infection. (CSV 65 KB)

Additional file 6: **Table S6.** Pathways related to the 186 genes that are highly over and under expressed for the HIV infection. (CSV 62 KB)

Additional file 7: **Table S7.** Phenotypic disease association for SARS infection based on the ICD9 codes at the 3-digit category level. Only statistically significant links with high relative risk *R* *R*_*ij*_ are considered. (CSV 9 KB)

Additional file 8: **Table S8.** Phenotypic disease association for SARS infection based on the ICD9 codes at the 5-digit category level. Only statistically significant links with high relative risk *R* *R*_*ij*_ are considered. (CSV 4 KB)

Additional file 9: **Table S9.** Phenotypic disease association for HIV infection based on the ICD9 codes at the 3-digit category level. Only statistically significant links with high relative risk *R* *R*_*ij*_ are considered. (CSV 8 KB)

Additional file 10: **Table S10.** Phenotypic disease association for HIV infection based on the ICD9 codes at the 5-digit category level. Only statistically significant links with high relative risk *R* *R*_*ij*_ are considered. (CSV 3 KB)

Additional file 11: **Table S11.** Highly statistical significant differentially expressed genes between SARS-CoV and reference group (Mock) in lung epithelial cells. (XLSX 20 KB)

Additional file 12: **Table S12.** Highly statistical significant differentially expressed genes between MERS-CoV and reference group (Mock) in lung epithelial cells. (XLSX 21 KB)

Additional file 13: **Figure S1.** Median expression profile of SARS-CoV vs Mock using hierarchical clustering (Ward method, Pearson correlation) of 215 statistical significantly differential expressed genes (*p*<0.001). The information regarding each of the clusters and genes is described in Additional file 11: Table S11. (PDF 8 KB)

Additional file 14: **Figure S2.** Median expression profile of MERS-CoV vs Mock using hierarchical clustering (Ward method, Pearson correlation) of 234 statistical significantly differential expressed genes (*p*<0.001). The information regarding each of the clusters and genes is described in Additional file 12: Table S12. (PDF 3 MB)

Additional file 15: **Table S13.** Connectivity Map results of predicted drugs per instance (for each drug and cells line) to reverse SARS-CoV for early and sustained signature (drugs with negative enrichment scores). (CSV 6 KB)
