# Supplementary figures and images for: Network-based analysis of comorbidities risk during an infection: SARS and HIV case studies
Source: BMC Bioinformatics. 2014 Oct 24;15(1):333. doi: 10.1186/1471-2105-15-333 (PMC4363349; doi:10.1186/1471-2105-15-333)

**Cluster 1**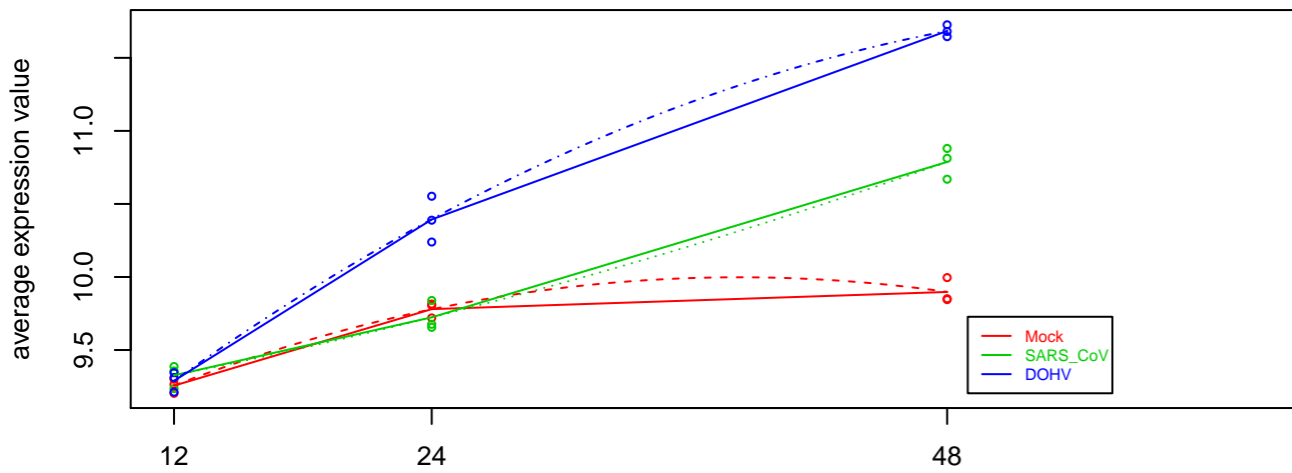**Cluster 2**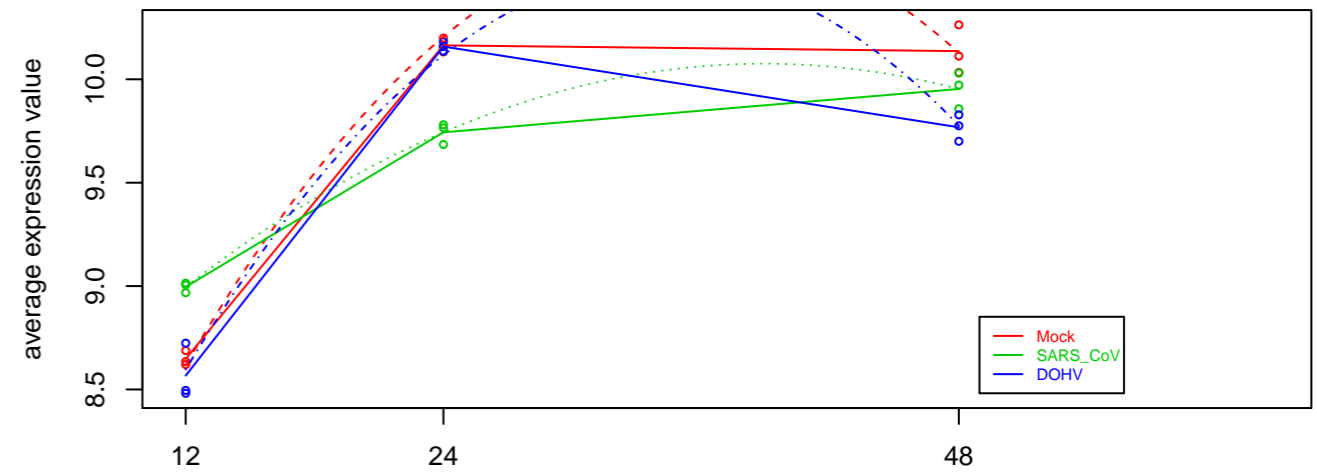**Cluster 3**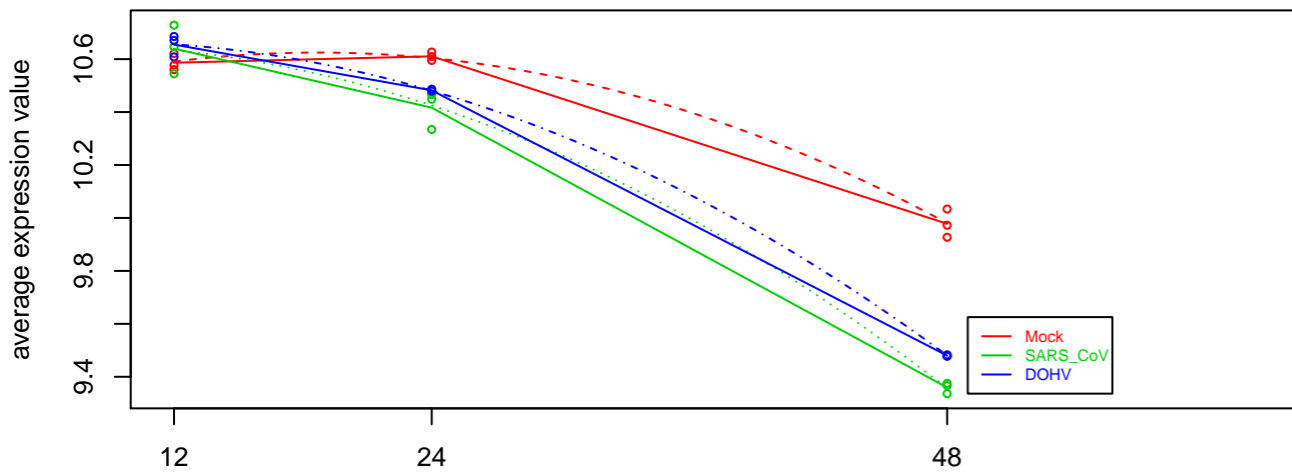

Supplement: Supplementary file 13 — Additional file 13: Figure S1. Median expression profile of SARS-CoV vs Mock using hierarchical clustering (Ward method, Pearson correlation) of 215 statistical significantly differential expressed genes (p<0.001). The information regarding each of the clusters and genes is described in Additional file 11: Table S11. (PDF 8 KB) [file 12859_2013_6782_MOESM13_ESM.pdf]

**Cluster 1**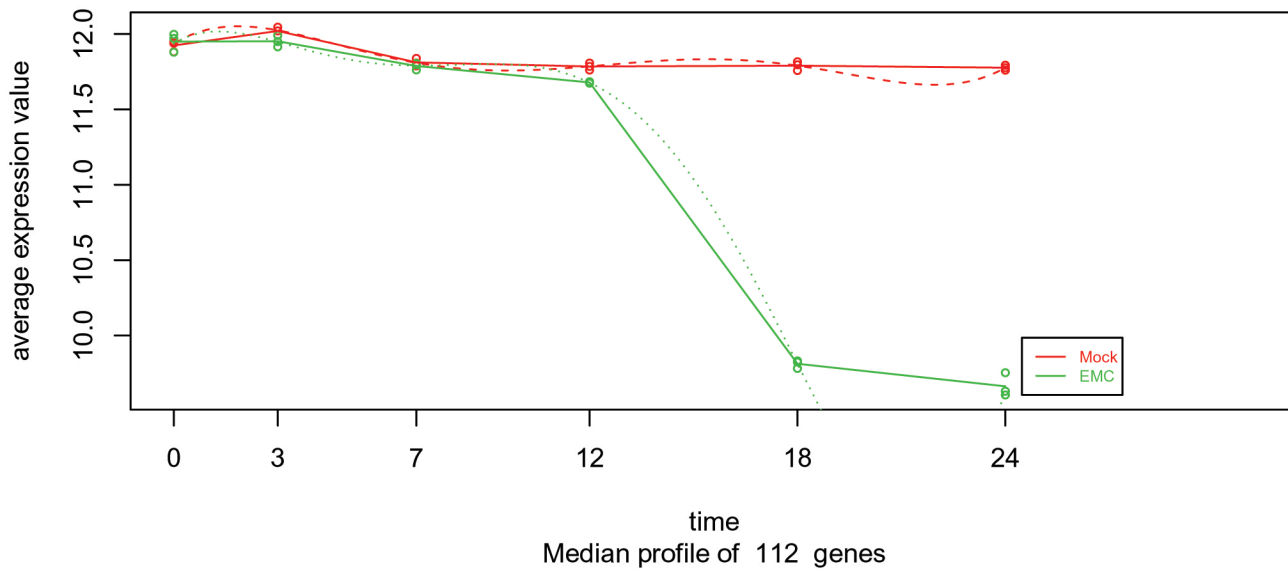**Cluster 2**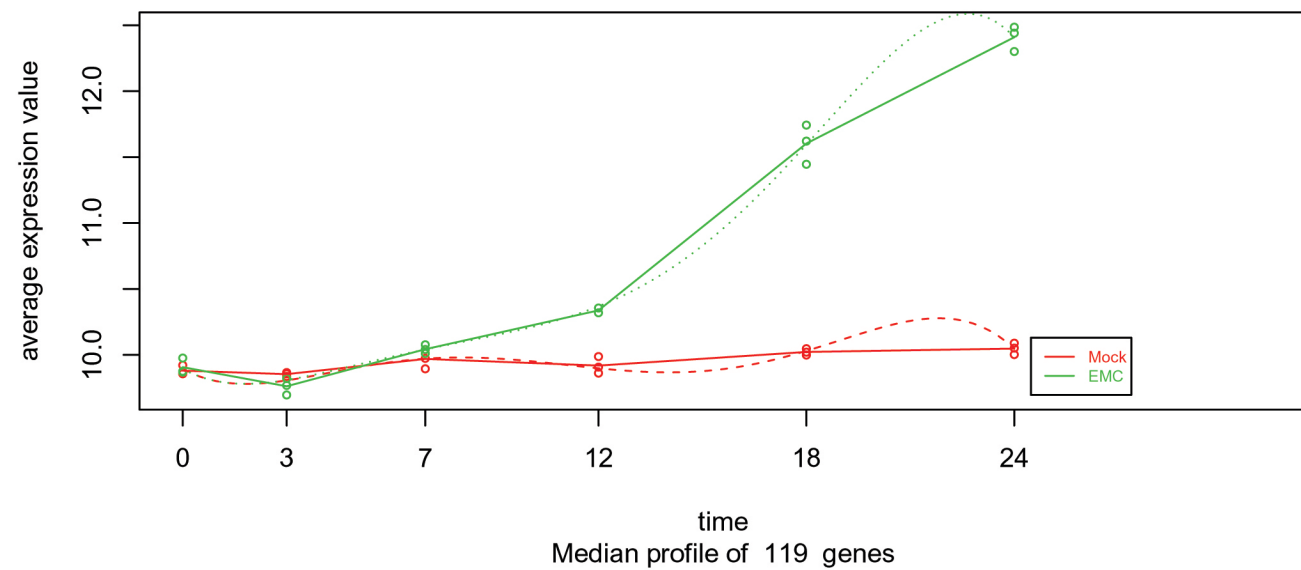**Cluster 3**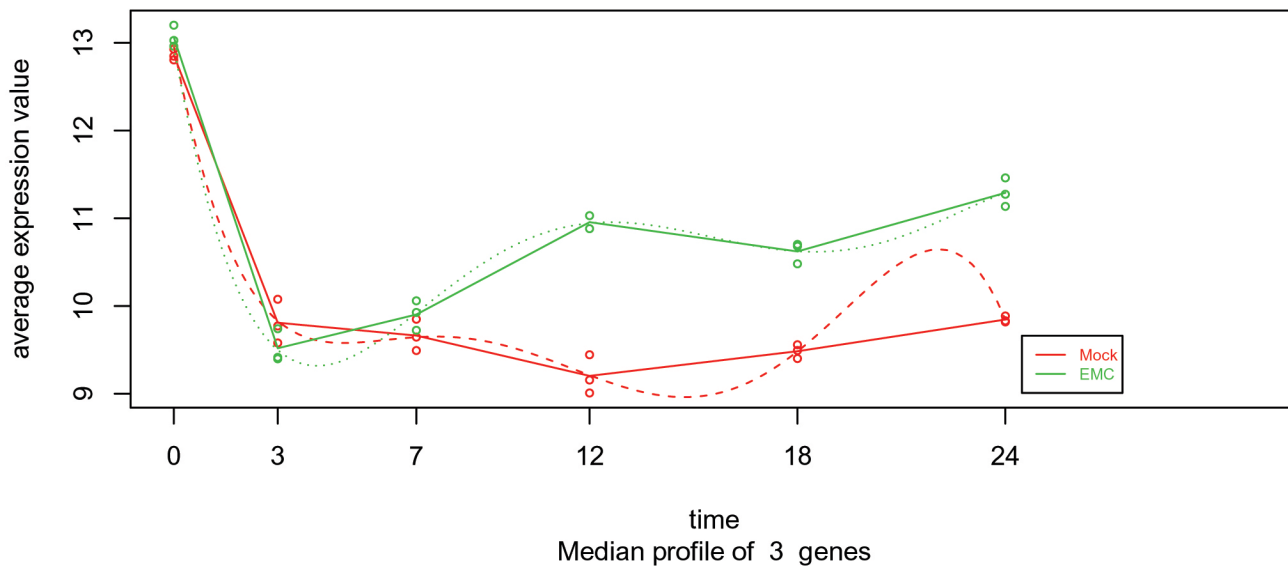

Supplement: Supplementary file 14 — Additional file 14: Figure S2. Median expression profile of MERS-CoV vs Mock using hierarchical clustering (Ward method, Pearson correlation) of 234 statistical significantly differential expressed genes (p<0.001). The information regarding each of the clusters and genes is described in Additional file 12: Table S12. (PDF 3 MB) [file 12859_2013_6782_MOESM14_ESM.pdf]
